# Prevalence and correlates of cesarean section delivery and physician attendance during cesarean section in Bangladesh: evidence from nationally representative data 2017–18

**DOI:** 10.1186/s12884-026-08992-8

**Published:** 2026-03-30

**Authors:** Sanjoy Kumar Chanda, Sk. Faijan Bin Halim, Ety Rani, Ashim Kumar Nandi, Meherab Hossain, Antora Rahut, Md. Ashfikur Rahman

**Affiliations:** 1 Sociology Discipline, Social Science School, Khulna University, Khulna, Bangladesh; 2 Economics Discipline, Social Science School, Khulna University, Khulna, Bangladesh; 3 Department of Sociology, Faculty of Social Sciences, University of Barishal, Barishal, Bangladesh; 4 Department of Gastroenterology, Shaheed Suhrawardy Medical College & Hospital, Dhaka, Bangladesh; 5 Development Studies Discipline, Social Science School, Khulna University, Khulna, Bangladesh

**Keywords:** Factors, Cesarean section, Childbirth, Physician attendance, Women, Bangladesh

## Abstract

**Background:**

Cesarean section (CS) rates in Bangladesh have increased dramatically from 3% in 2000 to 33% in 2017–18, substantially exceeding the recommended 10–15% range in global maternal health literature. While CS can be lifesaving when medically indicated, concerns exist regarding both overutilization and the qualifications of providers performing these surgical procedures. As CS is a major surgical procedure legally restricted to Bachelor of Medicine and Bachelor of Surgery (MBBS)-qualified physicians under Bangladeshi law, examining physician attendance distinct from the broader category of medically trained providers is critical for evaluating obstetric care quality and regulatory compliance. Therefore, this study aimed to identify the prevalence of CS delivery and physician-attended CS for women and its associated factors in Bangladesh.

**Methods:**

Data from the Bangladesh Demographic and Health Survey (BDHS) 2017–18 were analyzed for 3,650 women aged 15–49 years who delivered at health facilities within three years preceding the survey. ‘Physician attendance’ was identified using the BDHS question on delivery attendants. Respondents who reported ‘doctor’ (MBBS or higher qualification) as a delivery attendant were classified as having physician attendance. This is distinct from the broader BDHS category of ‘medically trained providers,’ which includes doctors, nurses, midwives, and other trained personnel. Binary logistic regression, guided by Andersen’s Behavioral Model, examined factors associated with (a) CS delivery and (b) Physician-attended CS. All analyses incorporated sampling weights to account for the complex survey design.

**Results:**

The prevalence of CS delivery was 33% (*n* = 1,741). Among CS deliveries, 24.3% (*n* = 423) had physician attendance. Factors significantly associated with CS delivery included geographic division, maternal age, antenatal care by physicians, birth order, education level (women and husbands), and wealth index. Similarly, regional location, maternal age, education, and wealth were significantly associated with physician-attended CS. Media exposure was associated with lower odds of CS delivery, but showed no significant association with physician-attended CS.

**Conclusions:**

This cross-sectional analysis identified significant sociodemographic gradients in both CS utilization and physician-attended CS. The findings suggest the need for improved facility reporting systems to distinguish medically indicated CS, strengthened regulatory oversight of private facilities, equitable deployment of qualified obstetric physicians in underserved regions, and evidence-based antenatal counseling on delivery options. Longitudinal research with clinical data is needed to establish causal pathways and inform targeted interventions.

## Introduction

A cesarean section (CS) is a surgical procedure to deliver a baby through incisions in the mother’s abdomen and uterus [7]. While CS can be lifesaving for mothers and infants when medically indicated, the World Health Organization recommends that population-level CS rates should not exceed 10–15% [12]. Global CS rates have risen substantially, reaching 21.1% worldwide during 2010–2018, with projections suggesting 28.5% by 2030 [13]. Bangladesh has experienced particularly dramatic increases, with CS rates rising from 3% in 2000 to 33% in 2017–18 [39].

CS delivery carries increased risks compared to vaginal birth, including maternal complications such as hemorrhage, infection, and adverse outcomes in subsequent pregnancies [27]. For infants, CS may affect microbiome development and has been associated with altered immune responses [42]. These health implications, combined with higher healthcare costs [20], underscore the importance of ensuring CS is performed only when medically indicated and by appropriately qualified physicians.

Bangladesh’s health policy framework explicitly addresses maternal health outcomes. The National Health Policy 2011 aims to reduce maternal and child mortality to rational levels [34], aligned with Sustainable Development Goal 3 targets to reduce maternal mortality to below 70 per 100,000 live births and neonatal mortality to at least 12 per 1,000 live births by 2030 [45]. However, the rapid expansion of CS in Bangladesh raises concerns about overutilization, as studies examining clinical indications have found that a substantial proportion of CS deliveries are performed without clear medical justification [4, 11].

CS is a major abdominal surgery requiring specialized training in obstetric surgical techniques, anesthesia management, and emergency complication response [38, 44]. Under the Bangladesh Medical and Dental Council (BMDC) Act 2010, only physicians with Bachelor of Medicine and Bachelor of Surgery (MBBS) or higher qualifications are legally authorized to perform surgical procedures [14]. This regulatory framework establishes the clinical and legal importance of examining provider qualifications for CS delivery. Despite this, Bangladesh's rapid private healthcare expansion has raised concerns about variability in provider qualifications and clinical standards across healthcare settings [1, 10].

Previous studies have examined sociodemographic factors associated with CS in Bangladesh, including maternal age, education, wealth, and place of residence [1, 3, 21, 29]. However, our review of the literature identified no studies using nationally representative data to specifically examine provider qualifications during CS delivery in Bangladesh. This gap is particularly significant given concerns about obstetric care quality in the country’s rapidly expanding but variably regulated private healthcare sector. Without understanding who receives physician-attended surgical delivery, it is difficult to assess whether rising CS rates reflect improved access to safe obstetric surgery or growing inequities in provider qualification. Therefore, this study aimed to identify the prevalence and correlates of CS delivery and physician attendance during CS in Bangladesh.

## Guiding framework

The Andersen Behavioral Model of Health Services Use [6] guided variable selection and analysis. This model posits that healthcare utilization is influenced by three categories of factors: predisposing factors (demographic and social factors that exist prior to illness), enabling factors (conditions that facilitate or impede access to care), and need factors (perceived and evaluated health status). The model has been widely applied in maternal health research in low- and middle-income settings [9].

**Fig. 1:**
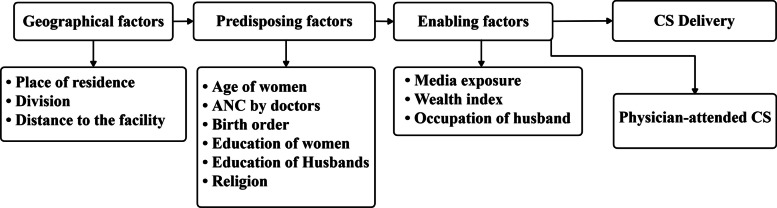
Conceptual framework adapted from Andersen’s Behavioral Model of Health Services Use [6]. The figure presents geographical, predisposing, and enabling factors examined in relation to cesarean section delivery and physician-attended CS in Bangladesh

For this analysis, we adapted this model to include three factor categories: geographical factors (place of residence, division, distance to facility), predisposing factors (age of women, ANC by doctors, birth order, education of women, education of husbands, religion), and enabling factors (media exposure, wealth index, occupation of husband). Need factors (medical indications for CS) were not included for two reasons: first, the Bangladesh Demographic and Health Survey (BDHS) does not comprehensively capture clinical indications, limiting the reliability of such measures; second, our focus was on sociodemographic and health system factors rather than clinical decision-making. We acknowledge that this analytical choice prevents distinguishing between medically indicated and potentially unnecessary CS. More broadly, the absence of clinical data, including medical indications for CS, obstetric complications, facility type, and provider availability, constrains interpretation of the appropriateness and quality of care associated with the observed patterns, a limitation discussed further below (Fig. 1).

## Methodology

### Study design and data source

This cross-sectional study analyzed data from the BDHS 2017–18, a nationally representative household survey conducted by the National Institute of Population Research and Training (NIPORT) with technical assistance from ICF International and funding from the U.S. Agency for International Development [39].

The BDHS 2017–18 employed a two-stage stratified cluster sampling design. The sampling frame comprised enumeration areas (EAs) from the 2011 Population and Housing Census conducted by the Bangladesh Bureau of Statistics. In the first stage, 675 EAs (250 urban, 425 rural) were selected with probability proportional to size across all eight administrative divisions. In the second stage, systematic samples of approximately 30 households per EA were selected. The survey interviewed 20,127 ever-married women aged 15–49 years from 19,457 households. For this analysis, we included 3,650 women who had facility-based deliveries within three years preceding the survey, as CS would only be possible in facility settings with appropriate infrastructure. Notably, restricting the sample to facility-based deliveries may introduce selection bias, as women delivering in facilities tend to be wealthier, more educated, and more urban than those delivering at home, potentially limiting generalizability.

### Variable description

#### Outcome variables

Two binary outcome variables were examined. First, CS delivery was assessed using the BDHS question: “Was the baby delivered by cesarean section; that is, did they cut your belly open to take the baby out?” (coded 0 = no, 1 = yes). Second, physician-attended CS was derived from the BDHS question ‘Who assisted with the delivery?’ which records all persons present during delivery using standardized DHS categories. CS deliveries where respondents reported ‘doctor’ as a delivery attendant were coded as 1, and others as 0. We use the term ‘physician-attended’ to refer specifically to MBBS-qualified doctors. This is distinct from the broader BDHS category of ‘medically trained providers’ or ‘skilled birth attendants,’ which includes doctors, nurses, midwives, paramedics, family welfare visitors (FWVs), community skilled birth attendants (CSBAs), and sub-assistant community medical officers (SACMOs). We focused specifically on physician attendance because CS is a major surgical procedure that only MBBS-qualified physicians are legally authorized to perform under the Bangladesh Medical and Dental Council Act.

It is important to note that the BDHS variable captures who “assisted with” or “attended” the delivery, not necessarily who performed the surgical procedure itself. Population-based surveys like DHS rely on women’s self-reports, but how women interpret this question and whether they accurately identify provider cadres is unknown [32]. Research has shown that women may have difficulty distinguishing between types of skilled providers when multiple attendants are present [41]. The 24.3% figure should therefore be interpreted as the proportion of CS deliveries where a physician was reported among attendants, acknowledging that this may not perfectly reflect who physically performed the surgery. This measurement limitation is discussed further in the limitations section.

#### Explanatory variable

The explanatory variables used in this study were based on the literature published on CS delivery in Bangladesh [11, 15, 21, 33, 35]. Based on Andersen’s behavioral model, the explanatory variables were categorized into three categories. Geographical factors included place of residence (urban/rural), division (Barishal/Chattogram/Dhaka/Khulna/Mymensingh/Rajshahi/Rangpur/Sylhet), and distance to the facility (no problem/problem). Predisposing factors included maternal age (12–19/20–35/36 years and above), antenatal care (ANC) by doctors (yes/no), birth order (1/2/3/4 and above), education of women and their husbands (no education/primary/secondary/higher), and religion (Muslim/others). Enabling factors included media exposure (yes/no), the wealth index (poorest/poorer/middle/richer/richest), and the occupation of husbands (professionals/business/manual jobs/unemployed).

#### Data analysis

Our analytical methodology consisted of three strategies: univariate, bivariate, and multivariate analyses, all of which were executed via the Statistical Package for Social Sciences, version 25 [22]. Descriptive statistics were used to describe the demographics of the sample population. Bivariate analysis was conducted via Pearson’s chi-square test to investigate the associations between the dependent and independent variables. The statistical significance was determined at a threshold of *p* < 0.05. To conduct the multivariate analysis, we used binary logistic regression models to find the parameters linked to CS delivery. All analyses incorporated DHS sampling weights to account for the two-stage stratified cluster sampling design, adjusting variance estimates for clustering at the enumeration area level and stratification by division and urban/rural residence. This approach ensures nationally representative estimates and appropriate standard errors for hypothesis testing. These models incorporated variables that exhibited statistical significance in the bivariate analysis. The strength of associations was estimated by using the adjusted odds ratio (AOR) with a 95% CI, and significance was declared at a *p-value* < 0.05. The logistic regression model can be expressed as follows:

i $$\mathrm{ln}\left(\frac{Pi}{1-Pi}\right)={\beta }_{0}+{\beta }_{1}{x}_{1}+\dots {\beta }_{m}{x}_{m}$$

where,* Pi* means probability and ${\beta }_{0}$, ${\beta }_{1},$…, ${\beta }_{m}$ are regression coefficients indicating the relative effect of a particular explanatory variable on the outcome variable. These coefficients change according to the context of the analysis in the study.

## Results

### Characteristics of the respondents

Table 1 reveals the demographic and socioeconomic profile of women who reported a recent childbirth. Most respondents resided in rural areas (65.8%), with regional concentration in Chattogram (16.7%) and Dhaka (14.7%), followed by Sylhet (14.4%), Mymensingh (11.9%), Rangpur (10.9%), Barishal (10.6%), Rajshahi (10.5%), and Khulna (10.3%). Approximately 60% reported that distance from the facility was a challenge in accessing healthcare facilities. Predisposing factors demonstrated that most women were aged 20–35 years (81.5%), with a few receiving ANC from doctors (18.2%). Women who experienced their first birth constituted the largest group (37.9%). Education levels varied, with nearly half having at least secondary education (47.8%), whereas 6.5% were illiterate. Most respondents were Muslim (91.7%). Enabling factors highlighted media exposure among two-thirds (67.1%) of women. The wealth distribution was relatively equal across all categories, with manual jobs being the predominant occupation for husbands (78.0%).

**Table 1:** CS Delivery and Physician-Attended CS by Sociodemographic Characteristics

Variables	Number (%) of women	% of CS delivery	*P* value	% of physician-attended CS	*P* value
Total	3650 (100.0)	1741 (33.0)		423 (24.3)	
Geographical factors					
Place of residence			<.001		<.000
Urban	34.2	57.0		50.4	
Rural	65.8	43.0		49.6	
Division			<.001		<.000
Barishal	10.6	11.6		10.9	
Chattogram	16.7	18.1		13.5	
Dhaka	14.7	12.2		22.0	
Khulna	10.3	8.5		13.9	
Mymensingh	11.9	12.8		8.7	
Rajshahi	10.5	9.7		8.3	
Rangpur	10.9	11.2		13.2	
Sylhet	14.4	16.0		9.5	
Distance to the facility			<.001		<.001
No problem	40.9	44.2		67.1	
Problem	59.1	55.8		32.9	
Predisposing factors					
Age of women			<.012		<.001
36 and above	1.2	1.2		0.9	
20–35	81.5	80.4		88.4	
12–19	17.4	18.4		10.6	
ANC by doctors			<.001		<.000
Yes	18.2	16.6		25.6	
No	81.8	83.4		74.4	
Birth order			<.001		<.000
1	37.9	34.8		51.5	
2	28.9	31.3		28.6	
3	16.9	18.5		14.4	
4 and above	16.4	15.5		5.4	
Education of women			<.001		<.000
Higher	17.8	10.3		45.6	
Secondary	47.8	47.3		44.4	
Primary	27.9	34.2		9.3	
No education	6.5	8.3		0.7	
Education of Husbands			<.001		<.000
Higher	19.2	11.1		48.8	
Secondary	32.9	32.0		32.5	
Primary	33.8	39.5		14.9	
No education	14.1	17.5		3.8	
Religion			<.021		<.090
Muslim	91.7	92.3		89.4	
Others	8.3	7.7		10.6	
Enabling factors					
Media exposure					
Yes	67.1	53.1	<.001	80.4	<.000
No	32.9	46.9		19.6	
Wealth index			<.001		<.000
Richest	20.2	11.1		48.5	
Richer	19.6	18.4		20.6	
Middle	18.0	18.5		14.9	
Poorer	20.6	23.8		10.4	
Poorest	21.7	28.1		5.7	
Occupation of husband			<.001		<.000
Professionals	11.2	5.8		34.6	
Business	8.3	9.8		4.4	
Manual jobs	78.0	82.0		58.7	
Unemployed	2.5	2.5		2.2	

### Prevalence of CS delivery and physician-attended CS

Table 1 indicates that 33% of facility-based deliveries in 2017–2018 were by CS, exceeding the 10–15% range historically referenced in global maternal health literature. Among CS deliveries, 24.3% of women reported a physician among delivery attendants. As noted in the Methods section, this figure reflects women’s recall of attendants present during delivery rather than verified surgical performance.

### CS delivery and its associated factors

A comparison of CS delivery and physician-attended CS reveals notable patterns in Bangladesh (columns 3 and 5 in Table 1). Chi-square analyses revealed significant associations between most explanatory variables and both outcomes.

Regarding geographical factors, urban women had higher rates of both CS delivery (57%) and physician-attended CS (50.4%) compared to rural women. The Chattogram division had a higher rate of CS delivery (18.1%), whereas the Dhaka division had a higher rate of physician-attended CS (22%). Distance to the facility showed contrasting patterns: it significantly prevented 55.8% of women from accessing CS, while a higher proportion of physician-attended CS (67.1%) occurred when access to the facility was unimpeded.

For predisposing factors, among CS deliveries, women aged 20–35 comprised the majority (80.4%), as did those among physician-attended CS cases (88.4%), consistent with this age group’s predominance in the overall sample. Women without ANC checkups constituted the majority of CS deliveries (83.4%) and physician-attended CS (74.4%). First-time mothers were strongly represented in CS deliveries (34.8%) and physician-attended CS (51.5%). Education patterns differed between outcomes: CS delivery rates were higher among women with secondary education (47.3%), whereas higher-educated women (45.6%) and women with higher-educated husbands (48.8%) were more frequently associated with physician-attended CS.

Concerning enabling factors, media exposure showed a parallel trend across both outcomes (53.1% for CS delivery and 80.4% for physician-attended CS). The wealth index demonstrated notable disparities: the poorest wealth quintile accounted for the largest proportion of CS deliveries (28.1%), likely reflecting the composition of facility-based births rather than higher CS rates among the poorest, whereas the wealthiest class (48.5%) more frequently had physician-attended CS. Women whose husbands had manual jobs reported higher rates of both CS delivery (82%) and physician-attended CS (58.7%).

## Multivariate analysis

### Factors associated with CS delivery

Table 2 shows that significant differences emerge in the estimation of factors associated with CS delivery. Geographically, women living in Dhaka (AOR: 1.478, 95% CI: 1.094–1.996) and Khulna (AOR: 1.877; 95% CI: 1.363–2.587) divisions presented significantly greater odds of CS delivery than those in Sylhet (reference category). Additionally, women aged 36 years and above presented higher odds (AOR: 2.423; 95% CI: 1.071–5.483) than younger women aged 12–19 years. Women who received ANC from doctors demonstrated higher odds (AOR: 1.264; 95% CI: 1.039–1.538) than those who did not; however, this association should be interpreted cautiously, as it may reflect physicians’ propensity to recommend CS, women with pregnancy complications preferentially seeking physician care, or unmeasured confounding. For birth order, first-time mothers (AOR: 2.237; 95% CI: 1.592–3.143) had notably greater odds than mothers with four or more children. The wealth index showed a significant association with CS delivery, with respondents in the richest wealth category having substantially higher odds (AOR: 4.009; 95% CI: 2.869–5.602) than those in the poorest category. Higher educational levels for women (AOR: 1.614; 95% CI: 1.011–2.577) and husbands (AOR: 1.844; 95% CI: 1.303–2.609) were associated with increased odds of CS delivery. Media exposure was significantly associated with lower odds of CS delivery (AOR: 0.666; 95% CI: 0.553–0.801). However, religion was not significantly associated with CS delivery.

**Table 2:** Binary logistic regression analysis to identify the factors associated with CS delivery and physician-attended CS

Factors	CS delivery				Physician-attended CS			
	AOR	(95% CI)		*P* value	AOR	(95% CI)		*P* value
		Lower	Upper			Lower	Upper	
Geographical factors								
Place of residence								
Urban	.957	.800	1.145	.632	.892	.664	1.198	.446
Rural (RC)	1				1			
Division								
Barishal	1.275	.903	1.800	.167	1.715	.968	3.037	.064
Chattogram	0.882	.653	1.193	.416	1.080	.634	1.840	.777
Dhaka	1.478	1.094	1.996	.011	1.663	1.005	2.754	.048
Khulna	1.877	1.363	2.587	.000	1.911	1.116	3.271	.018
Mymensingh	1.210	.871	1.679	.255	1.209	.672	2.178	.527
Rajshahi	1.543	1.113	2.140	.009	.975	.526	1.807	.936
Rangpur	1.211	.870	1.686	.257	1.893	1.103	3.250	.021
Sylhet (RC)	1				1			
Distance to the facility								
No problem	1.016	.865	1.193	.846	1.297	.991	1.699	.058
Problem (RC)	1				1			
Predisposing factors								
Age of women								
36 and above	2.423	1.071	5.483	.034	3.249	.933	11.308	.054
20–35	1.294	1.033	1.621	.025	1.663	1.097	2.521	.017
12–19 (RC)	1				1			
ANC by doctors								
Yes	1.264	1.039	1.538	.019	1.229	.911	1.657	.177
No (RC)	1				1			
Birth order								
1	2.237	1.592	3.143	.000	1.188	.664	2.125	.561
2	1.768	1.272	2.459	.001	.735	.410	1.315	.299
3	1.467	1.034	2.082	.032	1.063	.582	1.943	.842
4 and above (RC)	1				1			
Education of women								
Higher	1.614	1.011	2.577	.045	7.969	1.820	34.902	.006
Secondary	1.167	.769	1.769	.468	4.799	1.129	20.395	.034
Primary	.841	.552	1.281	.419	2.943	.684	12.662	.147
No education (RC)	1				1			
Education of Husbands								
Higher	1.844	1.303	2.609	.001	2.279	1.181	4.396	.014
Secondary	1.159	.868	1.547	.317	1.297	.705	2.384	.403
Primary	.999	.755	1.322	.993	.947	.512	1.752	.862
No education (RC)	1				1			
Religion								
Muslim	.780	.591	1.029	.079	.805	.533	1.217	.305
Others (RC)	1				1			
Enabling factors								
Media exposure								
Yes	.666	.553	.801	.000	.839	.602	1.169	.299
No (RC)	1				1			
Wealth index								
Richest	4.009	2.869	5.602	.000	3.082	1.675	5.671	.000
Richer	1.897	1.415	2.544	.000	1.639	.917	2.928	.095
Middle	1.854	1.399	2.458	.000	1.727	.980	3.045	.059
Poorer	1.364	1.039	1.790	.025	1.384	.788	2.432	.258
Poorest (RC)	1				1			
Occupation of Husband								
Professionals	1.086	.515	2.292	.828	.725	.275	1.914	.516
Business	1.073	.492	2.338	.860	.863	.284	2.619	.794
Manual jobs	.877	.427	1.799	.719	.550	.213	1.426	.219
Unemployed (RC)	1				1			

Here, *CI* Confidence Interval, *RC* Reference Category

### Factors associated with physician-attended CS

For physician-attended CS, the Dhaka (AOR: 1.663; 95% CI: 1.005–2.754) and Khulna (AOR: 1.911; 95% CI: 1.116–3.271) divisions remained significant, indicating greater odds in these regions (Table 2). Compared with younger women, women aged 20–35 years (AOR: 1.663; 95% CI: 1.097–2.521) reported significantly higher odds of physician-attended CS. The estimate for women aged 36 years and above (AOR: 3.249; 95% CI: 0.933–11.308, *p* = 0.054) did not reach statistical significance and exhibited a notably wide confidence interval reflecting smaller sample sizes; this estimate warrants cautious interpretation. Compared with women with no education, those with higher educational levels (AOR: 7.969; 95% CI: 1.820–34.902) and husbands with higher education (AOR: 2.279; 95% CI: 1.181–4.396) presented substantially increased odds of physician-attended CS; however, the wide confidence intervals indicate limited precision and potential instability of these estimates, likely due to smaller subsample sizes. These findings should therefore be interpreted as suggestive rather than definitive. Furthermore, women from the wealthiest families had considerably higher odds (AOR: 3.082; 95% CI: 1.675–5.671) than those in the poorest category. Media exposure was not significantly associated with physician-attended CS (AOR: 0.839; 95% CI: 0.602–1.169, *p* = 0.299).

## Discussion

The observed prevalence of CS delivery (33%) exceeds the 10–15% range often cited in maternal health literature [12], positioning Bangladesh among the highest CS-rate countries in South Asia. Although a substantial proportion of CS deliveries in Bangladesh may lack clear clinical justification [4, 11], our cross-sectional design cannot empirically verify whether specific CS deliveries were medically unnecessary. This rate surpasses those reported in India (21.5%, 2019–21) [36] and Pakistan (20% from 2017–18) [5], indicating that Bangladesh’s CS trajectory diverges from regional trends. Women who undergo CS delivery are more likely to experience physical complications such as hemorrhage, hysterectomy, transfusion, severe infection, shock, and uterine rupture [27, 30] and, in rare cases, maternal mortality [7]. Notably, only 24.3% of CS deliveries involved a reported physician attendant, raising questions about potential compliance with the BMDC Act 2010 requirement for physician-performed surgery [14], though this figure warrants careful interpretation given that the BDHS variable captures delivery attendance rather than verified surgical performance. Regardless of measurement precision, the substantial socioeconomic gradients in physician attendance documented here constitute the central empirical contribution, revealing structural inequities in who receives physician-led surgical care.

The geographic patterning of CS delivery and physician attendance reflects well-documented urban–rural disparities in obstetric care infrastructure across Bangladesh. Earlier studies conducted in Bangladesh and India have shown that a greater percentage of CSs are observed in urban areas than in rural areas [1, 3, 15, 21, 28]. The disparity in healthcare access between urban and rural areas likely reflects systemic inequities in the distribution of qualified physicians, infrastructure investment, and regulatory oversight. Rural health facilities in Bangladesh often operate with fewer specialists and limited specialist availability, whereas urban tertiary facilities concentrate on MBBS-qualified physicians [28]. This maldistribution means that rural women undergoing CS may face a structural disadvantage in accessing physician-led care, regardless of individual socioeconomic characteristics. These patterns parallel broader health workforce challenges documented across South Asia, where physician density in rural areas remains substantially below urban levels [2, 24]. Specifically, the regression analysis of this study indicated that the Dhaka and Khulna divisions show significantly higher odds of CS delivery (AOR: 1.478) and physician-attended CS (AOR: 1.911). These findings are consistent with those of prior studies [1, 16, 29] and suggest the necessity for targeted strategies to address the issues leading to variations in CS rates across regions.

Maternal age, ANC by doctors, and birth order each demonstrated independent associations with CS delivery, consistent with patterns observed across South and Southeast Asian settings [8, 19, 21, 25]. This study showed that women aged 36 years and above, compared with younger women, have significantly greater odds of CS delivery (AOR: 2.423). This pattern suggests that maternal age remains an important stratification variable in obstetric service planning. Additionally, women who receive ANC from doctors show higher odds of CS delivery (AOR: 1.264); however, this association should be interpreted cautiously as it may reflect physicians’ propensity to recommend CS, women with pregnancy complications preferentially seeking physician care, or unmeasured confounding rather than a direct causal effect. In contrast to the findings of a previous study [35], which indicated a greater chance of elective CS delivery for third or subsequent births, first-time mothers have a greater probability of undergoing CS delivery (AOR: 2.237). This divergence may reflect primigravid women’s apprehension about vaginal delivery, peer influence, or clinician tendency toward surgical intervention for first births in the absence of prior delivery history.

The education gradient in CS utilization and physician attendance merits particular attention. Both women’s and husbands’ higher education was independently associated with increased odds of CS delivery and physician-attended CS, corroborating previous findings [11, 26, 37]. To address these underlying factors, education and awareness programs concerning the advantages and disadvantages of CS delivery could be considered, particularly among less educated populations. Such programs should address documented misconceptions about vaginal delivery safety that influence CS preferences in Bangladesh. Additionally, incorporating these messages into ANC services can help provide continuity of information throughout pregnancy [17].

Household wealth emerged as among the strongest predictors of both CS delivery and physician attendance, with pronounced gradients across quintiles. Wealthier women, compared with the poorest, showed substantially higher odds of both CS delivery and physician attendance, consistent with wealth-CS associations documented in Bangladesh, India, Indonesia, Kenya, and Tanzania [18, 21, 23, 40, 43]. This wealth gradient likely reflects the concentration of physician-staffed facilities in the private sector, where out-of-pocket costs restrict access for lower-income women.

Higher media exposure was associated with lower odds of CS delivery (AOR: 0.666), a finding that diverges from prior research linking media to increased CS preference. However, media exposure was not significantly associated with physician-attended CS (AOR: 0.839, *p* = 0.299), suggesting differential pathways for these two outcomes. In line with the findings for CS delivery, Luce et al. [31] and Karim et al. [26] noted the media’s influence on childbearing preferences. This pattern suggests that media exposure may shape CS decision-making -potentially through increased awareness of surgical risks—without influencing access to physician-led care, which appears more strongly determined by structural and socioeconomic factors.

While several associations reached statistical significance, the magnitude and precision of estimates varied substantially. For example, the association between women’s higher education and physician-attended CS demonstrated a large effect size (AOR: 7.969) but with a wide confidence interval (1.820–34.902), indicating uncertainty in the exact magnitude of association. Conversely, wealth index estimates showed both strong magnitude and narrower confidence intervals, suggesting greater stability. These patterns underscore the importance of interpreting statistical significance alongside effect size and precision rather than relying solely on p-values.

## Strengths and limitations

This study has several strengths. First, to our knowledge, this is the first study using nationally representative data to examine factors associated with physician attendance during CS in Bangladesh, addressing an important gap in the literature on obstetric care quality. Second, the use of a large, nationally representative dataset (*n* = 3,650 facility-based deliveries) enhances the generalizability of the findings to the broader population of Bangladeshi women. Third, all analyses incorporated DHS sampling weights to account for the complex two-stage stratified cluster sampling design, ensuring appropriate variance estimation.

However, some limitations should be noted. First, the cross-sectional design limits temporal ordering between exposure variables and outcomes, preventing causal inference. Observed associations may reflect reverse causation or residual confounding. Second, the measure of “physician-attended CS” has important limitations. The BDHS variable captures who “assisted with” delivery rather than who physically performed the surgery. Multiple providers may be present during CS, and respondents may be uncertain about specific roles. The 24.3% figure should therefore be interpreted as the proportion of CS deliveries where a physician was reported among attendants, acknowledging that this may not perfectly reflect who physically performed the surgery. Third, the reliance on self-reported data introduces potential recall bias, particularly given the three-year reference period. Although delivery mode is generally considered a salient event that women recall reliably, reporting of provider cadre may be less precise. Fourth, BDHS lacks detailed clinical information such as obstetric indications for CS, pregnancy complications, facility type (public vs. private), and provider availability. As a result, we cannot differentiate medically necessary from potentially unnecessary CS, nor assess appropriateness or quality of surgical care. Fifth, several estimates exhibited wide confidence intervals (e.g., higher education AOR: 7.969, 95% CI: 1.820–34.902), warranting cautious interpretation despite statistical significance. Sixth, restricting the analysis to facility-based deliveries may introduce selection bias, as poorer and rural women are less likely to deliver in facilities. Consequently, findings may not be generalizable to all births in Bangladesh.

## Conclusions

The prevalence rates of CS delivery and physician-attended CS were 33% and 24.3%, respectively, in BDHS 2017–18. Several factors, such as place of residence, division, age, birth order, ANC by doctors, education, and wealth index, were consistently associated with both outcomes, with pronounced gradients in physician attendance. These findings have several policy implications. First, monitoring systems should distinguish between medically indicated and non-indicated CS through improved facility reporting mechanisms. Second, regulatory oversight of private facilities should be strengthened to ensure adherence to clinical guidelines. Third, expanding equitable access to qualified obstetric physicians in underserved regions may reduce socioeconomic disparities in surgical delivery care. Finally, antenatal counseling protocols should incorporate balanced, evidence-based information regarding delivery options to support informed decision-making. However, longitudinal and facility-linked data are required before designing targeted interventions.

## Data Availability

The BDHS 2017–18 data, which are publicly available, were used in this study. The data can be accessed at https://www.dhsprogram.com/Data/

## Abbreviations

ANC: Antenatal Care
AOR: Adjusted Odds Ratio
BBS: Bangladesh Bureau of Statistics
BDHS: Bangladesh Demographic and Health Survey
CI: Confidence Interval
EA: Enumeration Areas
CS: Cesarean Section
MBBS: Bachelor of Medicine and Bachelor of Surgery
RC: Reference Category
SDG: Sustainable Development Goal
